# The eyes have it: Alcohol‐induced eye movement impairment and perceived impairment in older adults with and without alcohol use disorder

**DOI:** 10.1111/acer.15509

**Published:** 2025-01-29

**Authors:** Nathan Didier, Dingcai Cao, Andrea C. King

**Affiliations:** ^1^ Department of Psychiatry & Behavioral Neuroscience University of Chicago Chicago Illinois USA; ^2^ Department of Behavioral and Social Sciences Brown University School of Public Health Providence Rhode Island USA; ^3^ Department of Ophthalmology and Visual Sciences University of Illinois at Chicago Chicago Illinois USA

**Keywords:** alcohol use disorder, eye movement, older adults, saccade, smooth pursuit, tolerance

## Abstract

**Background:**

While alcohol has been shown to impair eye movements in young adults, little is known about alcohol‐induced oculomotor impairment in older adults with longer histories of alcohol use. Here, we examined whether older adults with chronic alcohol use disorder (AUD) exhibit more acute tolerance than age‐matched light drinkers (LD), evidenced by less alcohol‐induced oculomotor impairment and perceived impairment.

**Method:**

Two random‐order, double‐blinded laboratory sessions with administration of alcohol (0.8 g/kg) or placebo. Participants (*n* = 117; 55 AUD, 62 LD) were 40–65 years of age. Eye tracking outcomes (pupil size, smooth pursuit gain, pro‐ and anti‐saccadic velocity, latency, and accuracy) were measured at baseline and repeated at peak and declining breath alcohol intervals. Participants rated their perceived impairment during rising and declining intervals.

**Results:**

Following alcohol consumption, older adults with AUD (vs. LD) showed less impairment on smooth pursuit gain and reported lower perceived impairment, but both groups showed similar pupil dilation and impairment on saccadic measures.

**Conclusions:**

While alcohol impaired older adults with AUD less than LD in terms of their ability to track a predictably moving object (i.e., smooth pursuit), both drinking groups were equally sensitive to alcohol‐induced delays in reaction time, reductions in velocity, and deficits in accuracy to randomly appearing objects (i.e., saccade tasks). Thus, despite decades of chronic excessive drinking, older adults with AUD exhibited similar oculomotor tolerance on pro‐ and anti‐saccade eye movements relative to their light‐drinking counterparts. Given that these individuals also perceived less impairment during intoxication, they may be at risk for injury and harm when they engage in real‐life drinking bouts.

## Graphical abstract

While chronic alcohol consumption may produce tolerance (less impairment) for certain behaviors, alcohol's effects on eye movements are unclear. In our comparison of older adults with chronic alcohol use disorder (AUD) and age‐matched light drinkers, the AUD group was less impaired in tracking a predictably moving object (smooth pursuit) but equally impaired in responding to randomly appearing targets (saccades), despite perceiving lower impairment. During real‐life drinking, impairment of saccadic movements may hinder their ability to mitigate dangers and threats.

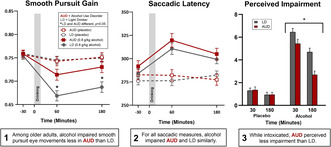

## INTRODUCTION

Alcohol intoxication is a leading cause for disease and injury, annually accounting for 7% of all U.S. emergency visits (National Institute on Alcohol Abuse and Alcoholism, 2024) and over 3 million deaths worldwide (World Health Organization, 2022). One reason for alcohol's propensity to cause harm is its acute effects on the oculomotor system. Alcohol inhibits central nervous system activity in regions like the cerebellum (Quinet & Goffart, 2015; Romano et al., 2017) and the lower anterior cingulate cortex (Marinkovic et al., 2013). Alcohol's pharmacodynamic properties may disrupt visual acuity via dilation of the pupil (Ma et al., 2011; Sanders, 2009) and a range of oculomotor deficits, that is, longer delays, reduced speed, and poorer accuracy of eye movements (Blekher et al., 2002; Fransson et al., 2010; Gale et al., 1996; Lehtinen et al., 1979; Roche et al., 2014; Tyson et al., 2021). These effects have been observed during eye tracking tasks, such as *fixation* (staring at a fixed target, enabling measurement of pupil size), *smooth pursuit* (tracking a moving object so that it remains on the fovea, the focal point of vision), *pro‐saccade* (rapid, ballistic movement toward an appearing object), and *anti‐saccade* (resisting a pro‐saccade and instead producing a saccade in the opposite direction) (Mahanama et al., 2022). Notably, some eye tracking tasks primarily involve “bottom up,” instinctive processes regulated by the superior colliculus and cerebellum (e.g., pro‐saccade; Robinson, 2022), while other eye tracking tasks also incorporate “top down,” conscious processes driven by cortical activity in the dorsolateral prefrontal cortex and frontal eye fields (e.g., smooth pursuit; Schröder et al., 2020).

Although drinking experience may result in behavioral tolerance over time, that is, a reduction in alcohol's acute impairing effects for certain fine motor, balance, and cognitive functions (Brumback et al., 2017; Didier et al., 2023; Elvig et al., 2021; Gatto et al., 1987; Wenger et al., 1981), the extent to which tolerance develops for oculomotor abilities is unclear (Maurage et al., 2020). Research in this area consists of two placebo‐controlled, double‐blinded studies conducted in our laboratory, and participants were limited to young adults (mainly in their 20s) who were light or heavy social drinkers (King & Byars, 2004; Roche & King, 2010). Results showed that an intoxicating dose of alcohol (0.8 g/kg) produced similar impairment in these subgroups on smooth pursuit and anti‐saccade tasks, but heavy drinkers showed less impairment on pro‐saccade measures, evidenced by shorter latency, greater velocity, and better accuracy compared with their lighter drinking counterparts. As the drinking histories of these young adults were 7–8 years on average, it is unclear if pro‐saccadic tolerance would also be evident in people with longer and more intense drinking patterns, such as middle‐ and older‐aged adults with alcohol use disorder (AUD).

Importantly, excessive drinkers often underestimate the impairing effects of alcohol (Love et al., 2023), and this may undermine the potential safeguarding role of tolerance. For instance, when blood alcohol concentration (BAC) is near the legal limit (0.08 g/dL), people with hazardous drinking patterns perceive less impairment than those with light drinking patterns even though they experience commensurate impairment on psychomotor and driving performance tests (Bernosky‐Smith et al., 2011; Brumback et al., 2017; Didier et al., 2023; Fillmore & Van Dyke, 2020; Roberts & Fillmore, 2017). A comparison between young adults with AUD who consumed a very high dose of 1.2 g/kg alcohol (resembling their typical drinking of 7–8 drinks) and age‐matched light drinkers who consumed a standard 0.8 g/kg dose (exceeding their typical drinking of 1–2 drinks) showed that the AUD group was more severely impaired than the LD group on fine‐motor and symbol‐matching tasks, but they perceived less impairment (Didier et al., 2023). Together, these studies demonstrate that perceptions of impairment are often blunted in young adult heavy social and AUD drinkers. Perceived impairment is clinically relevant given the heightened rates of injury among individuals with AUD (Rehm, 2011; Weil et al., 2018), as over‐confidence in their ability to operate a vehicle, socialize, or perform other duties when inebriated likely gives rise to high‐risk situations.

To our knowledge, drinkers with longer excessive drinking histories, such as those with AUD for 20+ years, have not been examined in studies assessing alcohol‐induced oculomotor changes and perceived impairment. Thus, the present study examined the acute effects of alcohol on oculomotor function and perception of impairment in older adults (aged 40–65 years) at several intervals across the breath alcohol curve. We assessed oculomotor function by administering tasks of fixation, smooth pursuit, pro‐saccade, and anti‐saccade, selected to capture both executive function (smooth pursuit, anti‐saccade) and automatic/reflexive action (fixation, pro‐saccade). Alcohol‐induced oculomotor impairment and self‐reported ratings of perceived impairment, and their associations, were compared between lifetime light social drinkers and excessive drinkers with chronic AUD. First, we hypothesized that alcohol would produce similar pupil dilation between groups, as prior research in alcohol‐dependent and control samples showed that both groups had similar pupillary constriction in response to a cholinergic muscle stimulant (methacholine) (Myers et al., 1979). Second, in line with prior results with young adult drinkers (Roche & King, 2010), we hypothesized that the AUD group would show less alcohol‐induced oculomotor impairment, especially in terms of pro‐saccadic function. Third, we hypothesized that the AUD group would perceive less impairment than the lighter drinkers and that both groups would show positive correlations between oculomotor deficits and perceived impairment ratings.

## METHODS

### Recruitment, screening, and participants

Study participants enrolled between December 2021 and December 2023 and were culled from the fourth cohort of the larger, ongoing Chicago Social Drinking Project. They were recruited via social media and public transport advertisements as well as word‐of‐mouth referrals. Based on initial telephone interviews, current non‐treatment seeking middle‐ and older‐aged adults (40–65 years) reporting no serious medical illnesses (liver or kidney disease, uncontrolled hypertension or diabetes, etc.) or medications (opiates, benzodiazepines, etc.) that may preclude alcohol administration were scheduled for an in‐person screening.

At the screening visit, study candidates completed informed consent and provided photo identification as well as breath, urine, and blood samples. Candidates also filled out a health survey, the Online Timeline Follow‐Back calendar for past‐month drinking (Rueger et al., 2012; Sobell & Sobell, 1992), the Alcohol Use Disorder Identification Test (Babor et al., 2001), and surveys about lifetime history of alcohol and substance use behaviors. A trained research assistant administered the non‐patient screening and AUD modules of the Structured Clinical Interview for the Diagnostic and Statistical Manual of Mental Disorders (SCID‐5; First, 2015). Exclusion criteria were: hepatic panel result (AST, ALT, or GGT) greater than three times the upper limit of normal range, systolic blood pressure >160 millimeters of mercury (mmHg) or diastolic >110 mmHg, eye disease (cataract, strabismus, amblyopia, glaucoma, age‐related macular degeneration, diabetic retinopathy), illicit drug use (cocaine, opioids, etc.) >3 times in the past month, cannabis and other substance use disorders (other than alcohol or tobacco), and, among pre‐ or peri‐menopausal women, a positive pregnancy test or desire to become pregnant in the next 3 months.

For inclusion, light drinkers (LD) had to consume 1–9 drinks per week as their predominant adult pattern and engage in infrequent binge drinking (less than monthly occasions with 5+ drinks for men or 4+ drinks for women). Participants in the AUD group had to meet past year criteria for AUD and report consuming 28 or more standard alcohol drinks weekly (21 for women) for at least the past 10 years and as the predominant pattern for most of their adult life.

### Procedure

The study was conducted at the Clinical Addictions Research Laboratory at the University of Chicago in testing rooms designed to resemble a living‐room like environment with a separate room for eye tracking tasks. Participants attended two double‐blinded 5‐h individual experimental sessions during which they consumed either a 0.8 g/kg alcohol beverage (target blood alcohol concentration [BAC] = 0.09 g/dL) or a placebo beverage in random order. Participants arrived midday (11:29 a.m. ± 45 min) and their first and second sessions were separated by at least 48 h.

Prior to each session, the participant was instructed to abstain from recreational drugs for 48 h, alcohol for 24 h, and caffeine, tobacco, and food for 3 h before arriving. They were also informed that they would need to provide a negative breathalyzer reading before each session and a negative urine toxicology test (cocaine, opioids, benzodiazepines, methamphetamines, amphetamines) administered randomly prior to one or both sessions. For pre‐ and peri‐menopausal women, negative pregnancy tests were also required at each visit. No participant had a positive breathalyzer, drug, or pregnancy test upon arrival.

Following these arrival breath and urine tests, the participant consumed a snack provided by the study that was ~20% of recommended daily calories (55% carbohydrates, 10% protein, and 35% fat; Schofield, 1985). After the snack, the participant then completed baseline measures, including eye tracking tests.

The beverage administration began at experimental time 0, in which the participant received two equal drink portions to consume for 5 min each, with a 5‐min rest interval between portions. During this time, the research assistant was present and engaged the participant in light conversation. Intended to have a novel appearance and flavor, the beverages were served in clear, plastic, and lidded cups with a straw and consisted of grape‐flavored beverage powder, sucralose‐based sweetener, water, and the appropriate dose of 190‐proof ethanol (16% volume alcohol, 0.8 g/kg). Women received an 85% dose to account for sex differences in body water (Frezza et al., 1990). The placebo dose was the same volume and contained 1% alcohol as a taste mask. To reduce alcohol expectancy, the participant was told that the beverage could contain a stimulant, sedative, alcohol, or a placebo, or a combination of two of these substances (Conrad et al., 2012). In addition to eye tracking, each timepoint included subjective surveys and psychomotor measures (outside the scope of this report).

### Eye tracking

At pre‐drink baseline (T0) and 60‐ and 180‐min after the onset of beverage consumption (T1 and T2, respectively), the participant was escorted nearby to a dimly lit room for 10–12 min of visual performance testing. Eye positions and pupil sizes from both eyes were recorded using an Eyelink 1000 Plus Eyetracker (SR Research, Ottawa, ON, Canada) with a resolution of 1000 Hz (1 recording per ms). The participant was seated at a small table and placed their head in a vertical forehead bar and chin rest to minimize head movement. Chair height and chinrest positions were adjusted for comfortable viewing of the stimuli on a 24″ Dell monitor, located 1 m in front of the participant. At each timepoint, a built‐in calibration procedure validated eye tracking precision (i.e., looking at targets located at center, right, left, top, and bottom of the display). After calibration, the research assistant launched the eye tracking program (a lab‐developed, Python‐based software) that included tasks in this order: *fixation* (for pupil size measurement), *smooth pursuit*, *pro‐saccade*, and *anti‐saccade* (see Video S1). Prior to each task, instructions were read aloud by the research assistant, the computer monitor displayed a corresponding demonstration, and the participant was asked to describe the task to ensure understanding.

### Fixation

To obtain pupil size, the first eye tracking task involved fixating at the center cross for 15 s while limiting blinking.

### Smooth pursuit

The smooth pursuit task consisted of watching a target (a red dot) move horizontally between 15° left and 15° right of the midline. The position of the target changed in a sinusoidal fashion, that is, it moved fastest at the midline and slowed down as it approached the 15° boundary on either side. The participant was instructed to follow the moving target as closely as possible, and to limit their blinking. At each timepoint (T0, T1, T2) there were three trials: the first at 0.1 Hz, the second at 0.2 Hz, and the third (and fastest) at 0.3 Hz. For each frequency, the target moved back‐and‐forth across the computer screen for four complete cycles. When then the target returned to the midline after its fourth cycle, the target disappeared, the program beeped to signal the end of trial, and the participant returned their gaze to the center fixation cross. After a brief rest (~5 s), the next trial began. In total, the smooth pursuit task took 73.3 s.

### Saccades

Next, the participant completed 14 trials of the pro‐saccade task followed by 14 trials of the anti‐saccade task. Before each saccade trial, the participant gazed at the fixation cross located at the center of the display. A series of 15 white evenly spaced dots extended to either side of the midline, with the farthest circle on each side located 15° from the midline. The trial began when a randomly selected dot turned red. For the pro‐saccade task, the participant was instructed to move their eye gaze toward the red dot as quickly as possible and to focus on it. For the anti‐saccade task, the participant was instructed to move their eye gaze to the *opposite* side of the red dot, equidistant from the midline. At the end of each pro‐saccade and anti‐saccade trial, the red dot disappeared and there was a beep to signal the participant to return their gaze back to the center fixation cross. The pro‐ and anti‐saccade tasks each took 60 s.

### Perceived impairment

At 30‐ and 180‐min post‐beverage consumption, the participant rated perceived impairment by completing a 4‐item questionnaire used previously by our group (Brumback et al., 2017; Didier et al., 2023). Each item was rated on a scale from 0 (not at all) to 10 (extremely): (1) “How impaired do you think you are at present? (problems with coordination or thinking),” (2) “How unsafe do you think it would be to drive an automobile at present?” (3) “If I were at work right now, others might think I was intoxicated or behaving unusually,” and (4) “If I had a chance now, I might be more likely than usual to do something I'd later regret.”

### Study discharge

At the end of each session, the research assistant verified that breath alcohol concentration (BrAC) ≤0.04 g/dL and requested a ride‐share service for participant transportation. After the second session, the participant was informed of their beverage assignment and debriefed. In the larger study, the participant then entered a 1‐week mobile ecological momentary assessment phase (outside the scope of this report). Upon completing all phases of the entire study, the participant was compensated $400 plus $25 if they arrived on time for their visits. All study details were reviewed and approved by the Institutional Review Board at the University of Chicago.

### Dependent variables

The dependent variable for the fixation task was *relative pupil size*. The Eyelink software returned the number of pixels associated with each pupil, which was an arbitrary value. Therefore, to standardize measurement across participants, we calculated pupil size changes relative to baseline (Tyson et al., 2021). Specifically, pupil size (in pixel counts) at T1 and T2 were each divided by the baseline pixel count, resulting in relative change scores at T1 and T2 for each participant at each session. As such, a relative pupil size of 1 indicates no change from baseline, greater than 1 indicates dilation, and less than 1 indicates contraction.

The dependent variable for smooth pursuit performance was *gain*, a measure of how closely the participants' eyes followed the target. Gain was calculated as the ratio between mean eye gaze velocity and mean target velocity, and these scores were averaged across all smooth pursuit frequencies. Smooth pursuit trials were deemed failures if latency >1 s or gain <0.50 (Wilson et al., 1993); 11.2% of trials met these criteria and were excluded from further analysis. Frequency of failed smooth pursuit trials did not differ between the LD and AUD groups (11.3% vs. 11.2%, *p* = 0.92).

The dependent variables for pro‐saccades and anti‐saccades were: *peak velocity*, the maximum speed (°/s) recorded during a trial, *latency*, duration (ms) between stimulus appearing and the initiation of the saccade, determined as the moment when eye movement velocity reached 30°/s, and *accuracy*, a measure of how closely eye gaze was from the target, calculated as 1 − abs (*D*_Error_/*D*_T_), where *D*_Error_ represents the distance between the participants' gaze and target and *D*_T_ represents the distance between the midline and target. Saccade trials were deemed failures if peak velocity > 1000°/s, latency > 1 s, or accuracy < 20% (Wilson et al., 1993). Using these criteria, 10.8% of pro‐saccade trials and 11.5% of anti‐saccade trials were excluded from further analysis, similar to fail rates in our prior work (12%–18%; Roche & King, 2010). Frequency of failed trials did not differ between groups for pro‐saccades (11.1% vs. 10.4%, *p* = 0.27) but they did differ for anti‐saccades (10.7% [LD] vs. 12.5% [AUD], *p* = 0.006), potentially due to inhibitory deficits associated with AUD (Si et al., 2022).

Finally, the dependent measure of perceived impairment was a mean composite score of the four perceived impairment items. At each timepoint and dose, the internal consistency coefficient of the four items supported a single underlying construct (*α* range: 0.72–0.86), consistent with factor analysis of this composite measure in our prior young adult sample (Didier et al., 2023).

### Statistical analyses

Sample characteristics were compared between groups using *t*‐tests for continuous variables and chi‐squared tests for categorical variables. BrAC was analyzed using a repeated measure ANOVA using only the alcohol session data (all values were 0.00 g/dL for the placebo session). To assess the convergent validity of saccadic and smooth pursuit measures, Pearson correlation tests were conducted using baseline data of the placebo session. For all main dependent variables, Generalized Estimation Equations (GEE) were used to analyze the effects of group, dose, and time and their interactions, while controlling for age, sex, and education. Significant GEE effects (*p* < 0.05) were further investigated with marginal linear contrast testing and Šidák‐corrected post‐estimation comparisons. In addition, Pearson correlation tests were used to assess the relationship between oculomotor impairment and perceived impairment. For these correlation analyses, only alcohol session data were used; oculomotor impairment was calculated as the average of the baseline change scores at T1 and T2 (i.e., [T0–T1 + T0–T2] ÷ 2) and perceived impairment was calculated as the average of the 30‐ and 180‐min ratings. Eye tracking and perceived impairment scores were analyzed in Stata 15.1 (College Station, TX, USA).

## RESULTS

### Sample characteristics

The sample consisted of 117 participants, with *N* = 62 in the LD group and *N* = 55 in the AUD group. Table 1 includes the main background and drinking characteristics for these groups. The groups did not differ on most background variables, except that the AUD group had fewer years of education than the LD group. The overall sample averaged 49.7 (±7.1 SD) years of age, 57% reported male sex assigned at birth, and 59% reported race as White, 29% Black, 8% more than one race, and 4% Other (Asian, Native American), with 10% of participants also reporting Hispanic/Latino ethnicity. As expected, participants with AUD had higher liver enzyme levels and heavier drinking patterns than LD across all quantity and frequency measures, AUDIT scores, and AUD symptom count (see Table 1). Of note, in the AUD group, the mean duration of probable lifetime AUD was 24.9 (±9.2 SD) years, confirming a predominant pattern of excessive drinking throughout their adulthood.

**TABLE 1:** Sample characteristics.

	LD (*N* = 62)	AUD (*N* = 55)	Significance (*p*)
Demographics			
Age (years)	50.5 (6.6)	48.9 (7.5)	0.22
Sex, male (*N*, %)	35 (56.4%)	32 (58.2%)	0.85
Race, white (*N*, %)	34 (54.8%)	35 (63.6%)	0.33
Ethnicity, Hispanic/Latino (*N*, %)	7 (11.3%)	5 (9.1%)	0.99
Education (years)	16.9 (2.2)	14.8 (2.5)	<0.001
Liver enzymes			
Alanine aminotransferase (ALT)	21.9 (10.0)	30.7 (20.3)	0.003
Aspartate aminotransferase (AST)	20.9 (6.0)	32.9 (24.1)	<0.001
Gamma‐glutamyl transferase (GGT)	24.6 (17.2)	40.1 (36.5)	<0.001
Drinking characteristics			
AUDIT total	3.4 (1.5)	15.5 (5.7)	<0.001
AUD symptoms (DSM‐5)	0.01 (0.1)	5.3 (2.4)	<0.001
Past month drinking			
Drinks per week	5.1 (3.9)	39.5 (19.8)	<0.001
Drinks per drinking day	2.0 (1.0)	7.5 (3.8)	<0.001
Percent days any alcohol drinking	29.3 (18.2)	78.2 (22.1)	<0.001
Percent days with heavy drinking^a^	1.7 (4.6)	63.2 (25.0)	<0.001
Percent days with high‐intensity drinking^b^	0.3 (2.5)	21.3 (27.5)	<0.001

*Note*: Data are means (SD) for continuous variables and *N* (%) for categorical variables.

Abbreviations: AUD, alcohol use disorder group; AUDIT, alcohol use disorder identification test; LD, light drinker group.

^a^ Heavy drinking is defined as a drinking occasion with 5+ drinks for a man or 4+ drinks for a woman.

^b^ High‐intensity drinking is defined as a drinking occasion with 10+ drinks for a man or 8+ drinks for a woman.

### Breath alcohol concentration

**FIGURE 1:**
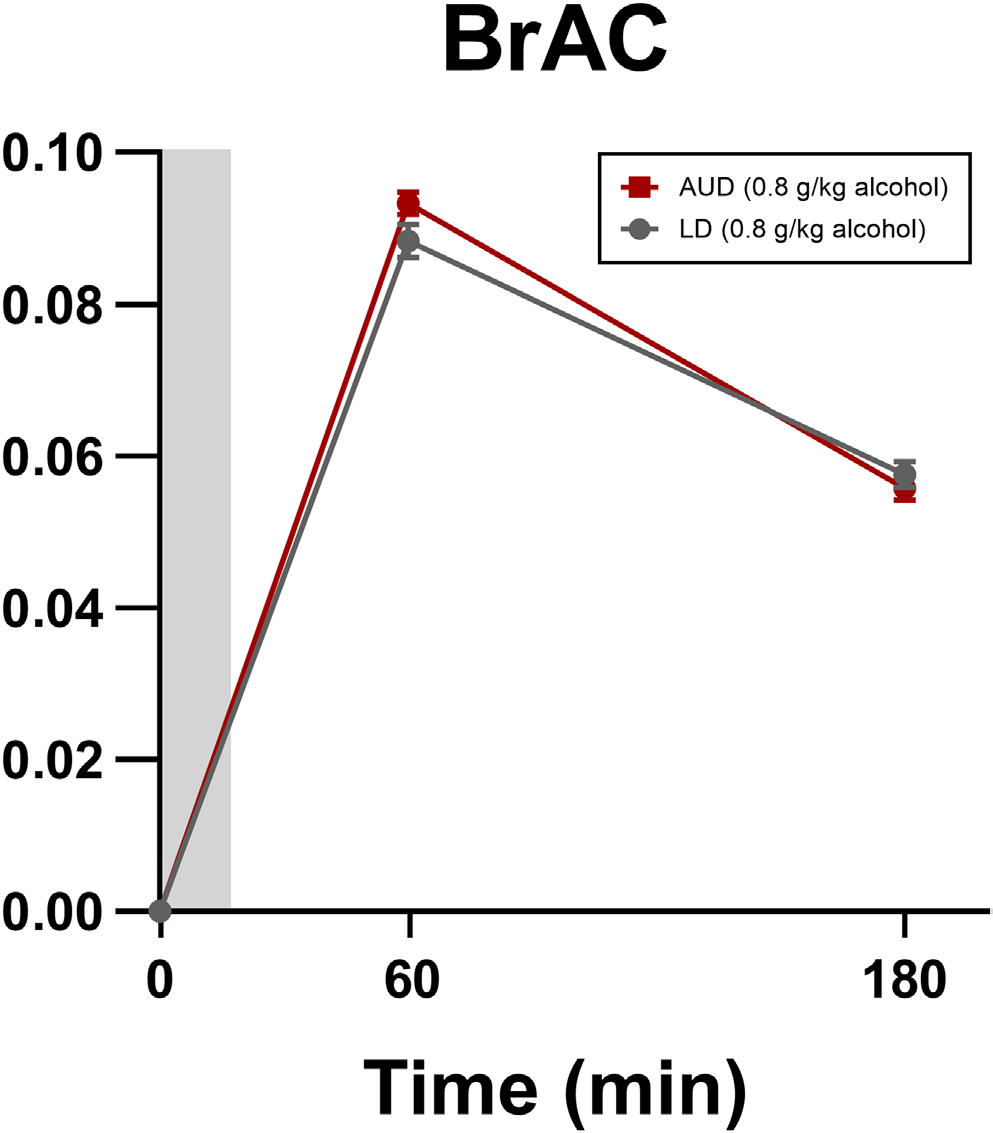
Data are mean ± standard error of breath alcohol concentration in AUD and LD during the alcohol session. The gray‐shaded region indicates beverage consumption. A group × time effect was observed (*p* < 0.05) such that AUD had higher levels than LD at T1 but not T2.

There was a two‐way group × time interaction for BrAC (*F*_1,107_ = 7.8, *p* = 0.006) such that the AUD group (vs. LD) had a higher BrAC at T1 (0.093 vs. 0.089 g/dL) but not at T2 (0.056 vs. 0.058 g/dL) (Figure 1).

### Pupil size

Table 2 displays GEE results of eye tracking outcomes. For pupil size, a two‐way dose × time interaction was observed such that alcohol increased pupil size from T0 to T1 but not from T1 to T2, and following placebo, pupil size increased in a time‐dependent manner (Table 2). Post‐estimation comparisons between sessions showed that alcohol produced more dilation than placebo at T1 (+17.5% vs. +8.6%, respectively; *p* < 0.001) but not at T2 (+18.1% vs. +14.2%, respectively; *p* = 0.57). Changes in pupil size did not differ between groups.

**TABLE 2:** GEE results of oculomotor performance.

Task	Variable	Dose × time			Group × dose × time			Post estimation	
		*χ* ^2^	df	*p*	*χ* ^2^	df	*p*	Alcohol session	Placebo session
Fixation	Pupil size	11.05	2	**0.004**	1.50	2	0.471	T1 > T0, T2 > T0	T2 > T1 > T0
Smooth pursuit	Gain	76.14	2	**<0.001**	12.22	2	**0.002**	T1 and T2: AUD > LD	ns
Pro‐saccade	Latency	23.27	2	**<0.001**	0.86	2	0.652	T1 > T2 > T0	ns
	Velocity	33.36	2	**<0.001**	1.28	2	0.528	T1 < T2 < T0	ns
	Accuracy	10.19	2	**0.006**	1.77	2	0.413	T1 < T2 < T0	ns
Anti‐saccade	Latency	11.12	2	**0.004**	2.66	2	0.265	T1 > T0, T1 > T2	ns
	Velocity	19.26	2	**<0.001**	1.47	2	0.48	T1 < T0, T2 < T0	ns
	Accuracy	0.74	2	0.69	3.31	2	0.191		

*Note*: Results from GEE analyses of dose (alcohol, placebo), time (T0, T1, T2), group (LD, AUD), and their interactions. Post‐estimation tests with *p* < 0.05 are bolded to indicate statistical significance. For dose × time effects, post‐estimation tests were conducted between timepoints within each session. For group × dose × time effects, post‐estimation tests were conducted between groups, within each session and timepoint. Smooth pursuit gain was the only variable to show an interaction effect with the group. All variables except anti‐saccade accuracy exhibited a dose × time interaction.

### Smooth pursuit and saccadic correlations at placebo baseline

Eye tracking performance at pre‐drink baseline was positively correlated between pro‐ and anti‐saccadic latency (*r* = 0.45), velocity (*r* = 0.61), and accuracy (*r* = 0.33; *p*s < 0.001). A positive relationship was also observed between pro‐saccadic accuracy and smooth pursuit gain (*r* = 0.45, *p* < 0.001), and, to a lesser extent, between anti‐saccadic latency and anti‐saccadic accuracy (*r* = 0.22, *p* = 0.02).

### Smooth pursuit performance

**FIGURE 2:**
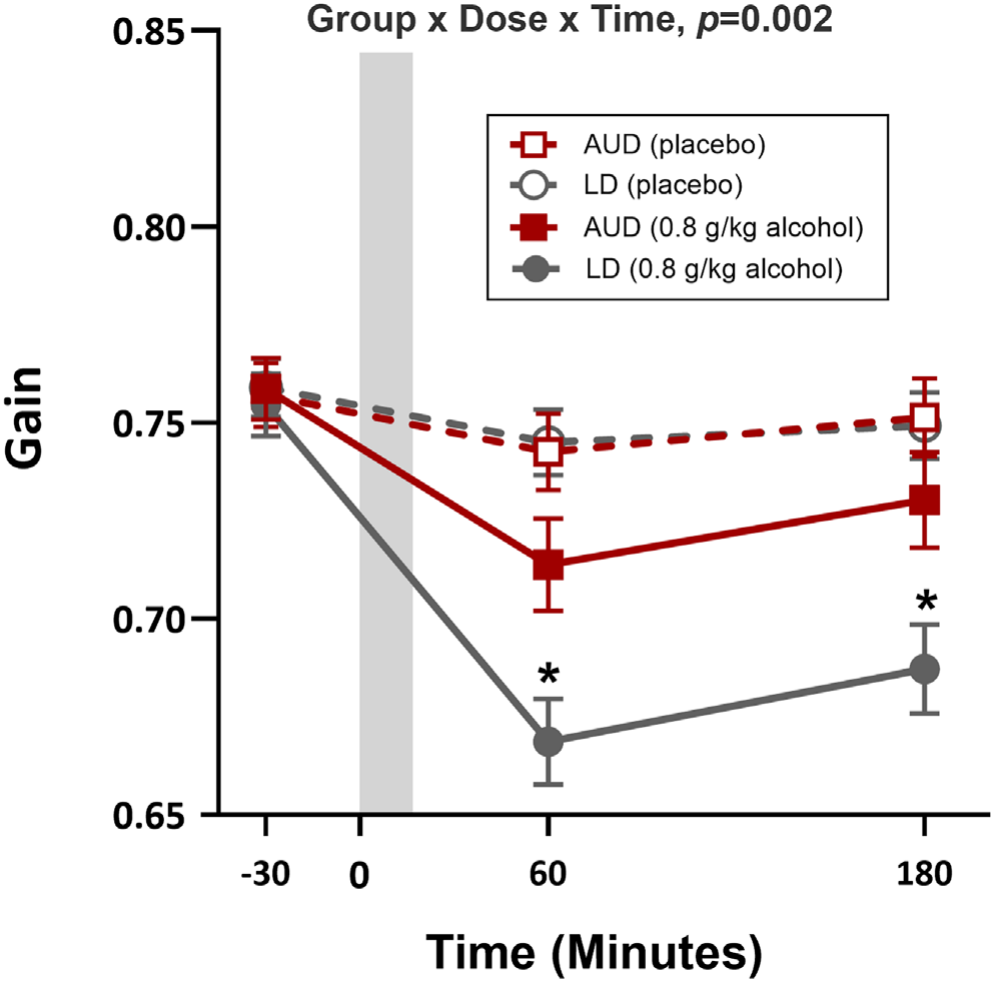
Data are mean ± standard error of gain at T0, T1, and T2 (−30, 60, and 180 min, respectively). The gray‐shaded region indicates beverage consumption. The AUD group showed less alcohol‐induced impairment than the LD group at T1 and T2. *AUD > LD, post‐estimation test within alcohol session, *p* < 0.001.

For smooth pursuit gain, a three‐way group × dose × time interaction was observed such that alcohol produced more impairment in LD than AUD at both post‐drinking timepoints (see Table 2, Figure 2). Relative to the baseline, alcohol reduced gain by 11% at T1 and 9% at T2 for the LD group, versus 6% at T1 and 4% at T2 for the AUD group.

### Pro‐saccadic performance

**FIGURE 3:**
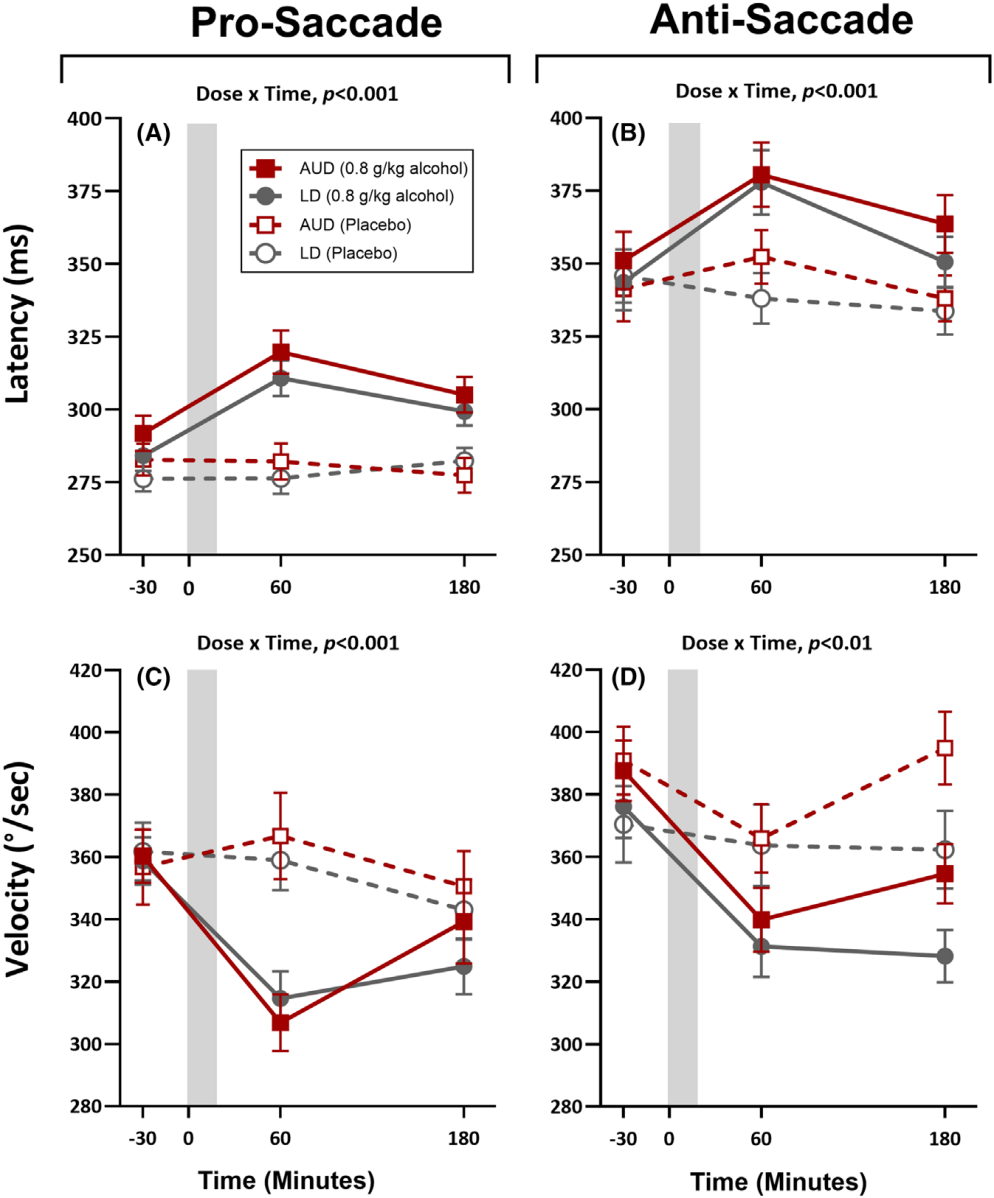
Data are mean ± standard error of saccadic latency and velocity at T0, T1, and T2 (−30, 60, and 180 min, respectively). The gray‐shaded regions indicate beverage consumption. Each variable showed a dose × time effect (*p*s < 0.01) such that alcohol impaired performance while placebo did not. No differences were observed between the LD and AUD groups.

In terms of pro‐saccadic latency, velocity, and accuracy, two‐way dose × time interactions were observed such that alcohol produced impairment at both T1 and T2 with the largest impairment occurring at T1, and no differences between groups (Table 2, Figure 3A,C). Compared with the AUD group, the LD group showed greater reductions in pro‐saccadic accuracy, but this was independent of dose (i.e., not an effect of alcohol).

### Anti‐saccadic performance

In regard to anti‐saccadic performance, alcohol impaired latency at T1 but not T2, (Figure 3B) and alcohol impaired velocity at both T1 and T2 (Figure 3D). The groups did not differ on either of these anti‐saccade performance measures (see Table 2). Alcohol did not impair anti‐saccadic accuracy.

### Perceived impairment

**FIGURE 4:**
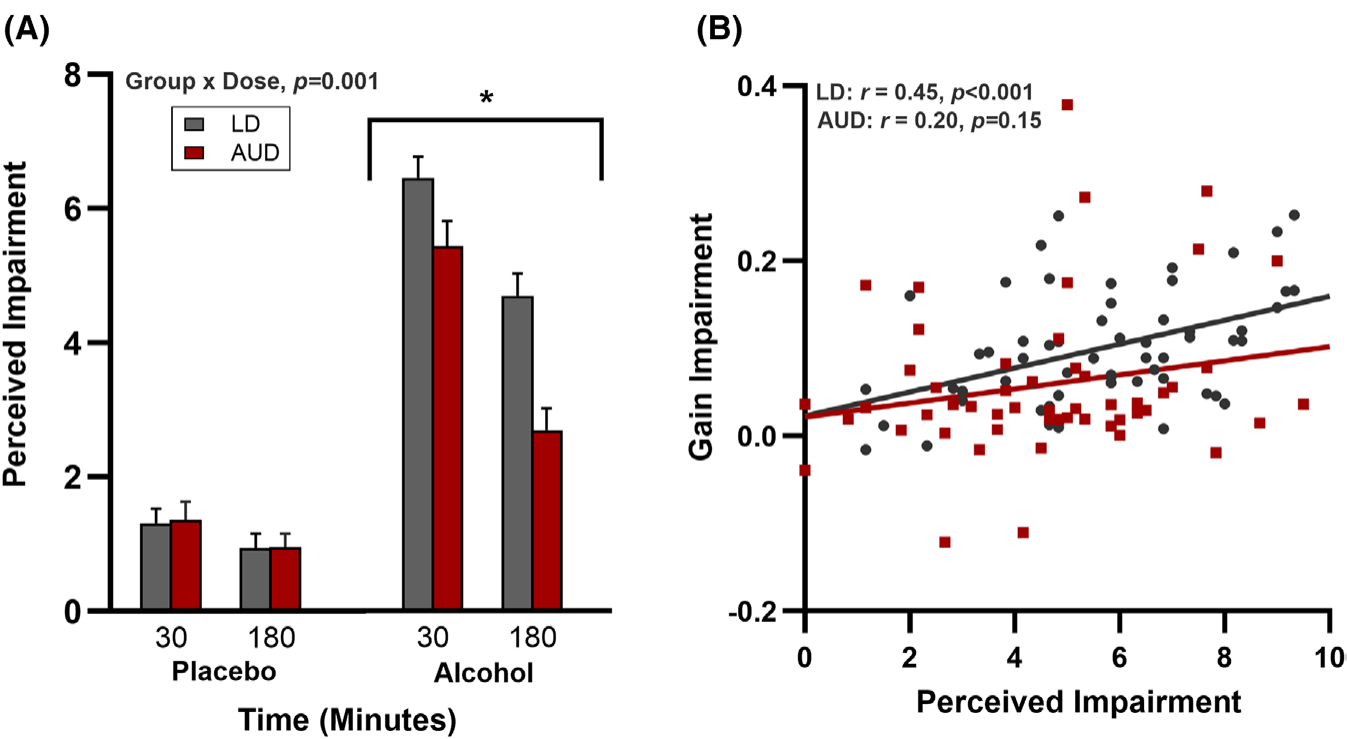
(A) Data are mean ± standard error of perceived impairment following either placebo (left) or alcohol (right). Following alcohol consumption, the LD group perceived more impairment than the AUD group. (B) Data are scores of perceived impairment (average of the 30‐ and 180‐min intervals) and gain impairment (average of the baseline change scores at T1 and T2, that is, [T0–T1 + T0–T2] ÷ 2) during the alcohol session, with linear regression fit lines for each group. The positive correlation within LD indicates that increases in their perceived impairment were associated with more impairment in their smooth pursuit performance. *AUD < LD, *p* < 0.01.

Figure 4 depicts the perceived impairment results. Alcohol produced greater perceived impairment ratings than placebo at both the 30‐ and 180‐min intervals, with larger perceived alcohol impairment at 30 min than 180 min (dose × time, *χ* ^2^_(1)_ = 19.3, *p* < 0.001). The LD group perceived more alcohol impairment than the AUD group (group × dose, *χ* ^2^_(1)_ = 9.7, *p* = 0.002; Figure 4A). Perceived impairment ratings during the alcohol session were positively correlated with smooth pursuit gain impairment (*r* = 0.35, *p* < 0.001) and, by examining correlations separately for each group, this appeared to be driven more by the LD group (*r* = 0.45, *p* < 0.001) than the AUD group (*r* = 0.20, *p* = 0.15) (Figure 4B).

## DISCUSSION

The present study is the first to examine alcohol‐induced eye movement effects, pupillary responses, and perceived impairment in middle‐to‐older adults with AUD. With a chronic AUD sample averaging nearly 40 drinks per week, this study examined individuals with more excessive drinking patterns than in prior studies of alcohol‐induced oculomotor impairment (e.g., young adults who averaged 20 drinks per week; Roche & King, 2010). By comparing older adults who have AUD with similarly aged light drinkers (LD), we observed several important findings. First, for both the LD and AUD groups, alcohol dilated pupil size more than placebo during the early testing phase. Second, the AUD drinkers (vs. LD) showed less alcohol‐induced impairment on smooth pursuit throughout testing but both groups showed similar impairment on the pro‐ and anti‐saccade measures. Third, the AUD group perceived less impairment during intoxication than LDs, and, especially for the LD group, perceived impairment was positively correlated with smooth pursuit impairment. Thus, the AUD group exhibited greater oculomotor tolerance only for smooth pursuit but not for reflexive saccades or pupillary responses. This is of importance as individuals with decades of AUD perceived they were less impaired than their light‐drinking peers, yet an intoxicating dose of alcohol (somewhat lower than what they typically consume) produced similar impairments in the latency, velocity, and accuracy of their saccadic movements. Individuals with AUD may believe they can “hold their liquor” and perform well when inebriated (Didier et al., 2023), but this perception is incongruous with objective measures showing that they are susceptible to oculomotor impairment. Such over‐confidence may underlie the high rates of alcohol‐associated injury and harm observed in excessive drinkers (Rehm, 2011; Taylor & Rehm, 2012; Weil et al., 2018).

To delineate why tolerance was evident for smooth pursuit but not saccades, it is important to consider the underlying neurobiology of these eye movements. The smooth pursuit task involves prolonged visual focus on a predictably moving target, activating brain regions known for their plasticity such as the visual cortex and frontal lobe (Grossberg et al., 2012; Schröder et al., 2020). Surprisingly, the visual cortex shows greater capacity for white matter reorganization at older ages, as was illustrated by comparisons in neuro‐imagining results between young adults (in their 20s) and senior adults (in their 70s) after visual processing training (Yotsumoto et al., 2014). With plasticity at advanced ages, smooth pursuit neurobiology may gradually adapt to alcohol over decades of drinking. This might explain why differential smooth pursuit tolerance was observed in older adults but not in young adults with shorter drinking histories (King & Byars, 2004; Roche & King, 2010).

In contrast, saccades are reflexive, ballistic eye movements in response to unpredictably appearing targets, and these eye movements activate subcortical regions with minimal plasticity (Grossberg et al., 2012; Robinson, 2022). In the present study, the LD and AUD groups, when challenged with a 0.8 g/kg dose of alcohol (4–5 standard drinks), showed similar alcohol‐induced impairments of saccadic latency, velocity, and accuracy. This finding contrasts with our prior research showing that young adult heavy drinkers versus light drinkers are tolerant to (i.e., show less) pro‐saccadic impairment (Roche & King, 2010). Thus, there may be differences in the development of tolerance in young adulthood compared to middle‐ and older‐adulthood, and also based on more excessive drinking in AUD versus that of heavy social drinkers (40 vs. 20 drinks per week). Importantly, older drinkers with AUD are sensitive to alcohol‐induced impairment of saccadic movements, and this corresponds to slower responses to environmental signals and unexpected objects (Bannerman et al., 2009; Srivastava et al., 2018). Given their high frequency of excessive drinking bouts (two‐thirds of days) and their susceptibility to saccadic impairment, they may be at heightened risk of injury, as nearly half of patients treated for traumatic brain injury meet criteria for AUD (Weil et al., 2018) and fatal vehicle injuries are more than 10 times as likely during binge episodes (Taylor & Rehm, 2012).

As already noted, the older drinkers with AUD perceived less impairment during intoxication than the light drinkers, and this corroborates prior findings in young adults (Brumback et al., 2007, 2017; Didier et al., 2023; Love et al., 2023). We also found that greater perceived impairment was correlated with poorer smooth pursuit performance but not correlated with saccadic performance. This supports the notion that smooth pursuit is a more conscious activity than saccadic movements (Spering & Carrasco, 2015; van Zoest & Donk, 2010; Zhaoping, 2012). Notably, the correlation between perceived impairment and smooth pursuit impairment was stronger for the LD than AUD group, suggesting that individuals with chronic AUD may have less awareness of their actual impairment. This finding echoes a takeaway from our prior study: despite confidence in their ability to operate a vehicle, socialize, or perform work duties, those with AUD remain sensitive to alcohol‐induced impairment, as evidenced by psychomotor deficits in young adults (Didier et al., 2023) as well as saccadic deficits in older adults.

The present study had several notable strengths and limitations. In addition to a robust, placebo‐controlled, and double‐blinded laboratory design with a fixed intoxicating alcohol dose, this study provided insight into alcohol effects in older adults aged 40–65 years, a population that is underrepresented in the field. Regarding limitations, only one alcohol and one placebo laboratory session were undertaken, alcohol was administered in a fixed oral dose at 0.8 g/kg, and eye tracking was conducted at two post‐ingestion intervals without neuroimaging. Therefore, analyses of oculomotor impairment were constrained in terms of providing a fuller picture of potential dose–response, temporal, and neurobiological effects, as well as direct effects of oculomotor impairment on injury risk (which could potentially be elucidated via ecological momentary assessment). Further, the study employed a cross‐sectional design that enabled comparisons between older individuals with and without AUD. However, without a longitudinal design to examine the relationship between aging and oculomotor tolerance, the possibility that the observed group differences were premorbid cannot be completely ruled out. Finally, the perceived impairment scale assessed a general array of impairments (social, vehicular, work, regret) that formed one factor index, but this scale did not specifically ask about perceptions of visual acuity or eye movements, and this limits direct conclusions about whether participants can perceive oculomotor impairment.

In conclusion, older drinkers are acutely impaired by alcohol in terms of eye movement reaction time, speed, and accuracy. Those with AUD (vs. LD) showed greater tolerance regarding smooth pursuit performance, but no differences were observed between groups for pro‐saccades, anti‐saccades, and pupil dilation. With decades of excessive drinking experience, individuals with chronic AUD perceived less impairment than LD, but they were still vulnerable to alcohol‐induced impairment of eye movements, that is, delayed initiation, slower velocity, and poorer accuracy. Therefore, older adults with chronic AUD are likely prone to injury when they engage in real‐life drinking bouts, as their ability to detect and respond to unexpected objects is hindered during intoxication.

## CONFLICT OF INTEREST STATEMENT

Authors have no declarations of competing interests to declare.

## Supporting information


Video S1

## Data Availability

The data that support the findings of this study are available from the corresponding author upon reasonable request.
